# (*E*)-1,2-Bis(1-allyl­benzimidazol-2-yl)ethene

**DOI:** 10.1107/S1600536810007890

**Published:** 2010-03-06

**Authors:** Li-Zhuang Chen

**Affiliations:** a School of Material Science and Engineering, Jiangsu University of Science and Technology, Zhenjiang 212003, People’s Republic of China

## Abstract

In the title compound, C_22_H_20_N_4_, the two benzimidazole ring systems are nearly coplanar [dihedral angle = 4.70 (5)°]. Two terminal C atoms of one allyl group are disordered over two sites of equal occupancy. The crystal structure is stabilized by π–π stacking inter­actions, the centroid–centroid distance between nearly parallel [dihedral angle = 19.82 (4)°] benzene and imidazole rings being 3.7885 (15) Å.

## Related literature

For the properties of bis­(imidazole) compounds, see: Knapp *et al.* (1990 ▶); Stibrany (2001 ▶); Stibrany *et al.* (2002 ▶).
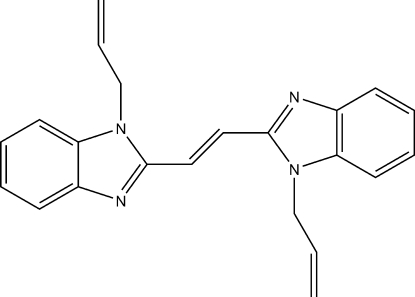


## Experimental

### 

#### Crystal data

C_22_H_20_N_4_
*M* *_r_* = 340.42
Monoclinic, 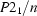

*a* = 11.008 (2) Å
*b* = 13.884 (3) Å
*c* = 12.540 (3) Å
β = 106.98 (3)°
*V* = 1833.2 (6) Å^3^
*Z* = 4
Mo *K*α radiation
μ = 0.08 mm^−1^
*T* = 293 K
0.30 × 0.25 × 0.22 mm

#### Data collection

Rigaku SCXmini diffractometer
18577 measured reflections
4190 independent reflections
2460 reflections with *I* > 2σ(*I*)
*R*_int_ = 0.061

#### Refinement

*R*[*F* ^2^ > 2σ(*F* ^2^)] = 0.058
*wR*(*F* ^2^) = 0.149
*S* = 1.03
4190 reflections
254 parameters
H-atom parameters constrained
Δρ_max_ = 0.15 e Å^−3^
Δρ_min_ = −0.17 e Å^−3^

### 

Data collection: *CrystalClear* (Rigaku, 2005 ▶); cell refinement: *CrystalClear*; data reduction: *CrystalClear*; program(s) used to solve structure: *SHELXS97* (Sheldrick, 2008 ▶); program(s) used to refine structure: *SHELXL97* (Sheldrick, 2008 ▶); molecular graphics: *ORTEP-3 for Windows* (Farrugia, 1997 ▶); software used to prepare material for publication: *SHELXL97*.

## Supplementary Material

Crystal structure: contains datablocks I, global. DOI: 10.1107/S1600536810007890/xu2721sup1.cif

Structure factors: contains datablocks I. DOI: 10.1107/S1600536810007890/xu2721Isup2.hkl

Additional supplementary materials:  crystallographic information; 3D view; checkCIF report

## Figures and Tables

**Fig. 1.**
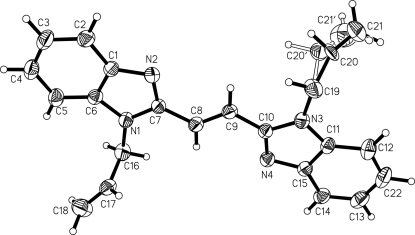
The molecular structure of the title compound with atom labels. Displacement ellipsoids were drawn at the 30% probability level. One disordered component is omitted for clarity.

**Fig. 2.**
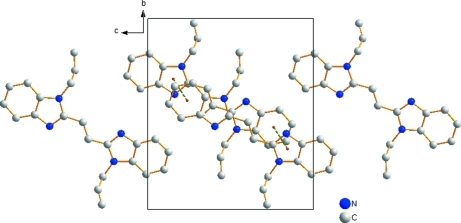
The unit cell packing diagram showing π-π stacking between benzene and imidazole rings. H atoms have been omitted for clarity.

## supplementary crystallographic information

### Comment

Recently, much attention has been devoted to compounds containing bis(imidazoles) due to their interesting properties, such as electron self-exchange (Knapp *et al.*, 1990), catalysts (Stibrany, 2001), and proton sponges (Stibrany *et al.*, 2002). In our laboratory a compound containing bis(imidazoles) has been synthesized, its crystal structure is reported herein.

In the title compound, C_22_H_20_N_4_, the benzimidazole moieties are essentially planar; two allyl groups are not on the planar. The atoms C20 and C21 of terminal olefin show disorder. The crystal structure is stabilized by π-π stacking between benzimidazolium units [the centroid-to-centroid distances between stacking benzene rings and imidazole are 3.7885 (15) Å.

### Experimental

Under N_2_ atmosphere, NaH (60 mmol, 1.44 g) was added to a mixture of (*E*)-1,2-bis(benzimidazol-2-yl)ethene (10 mmol, 2.6 g ) dimethylformamide (30 ml). After a reaction time of 20 min, the appropriate allyl bromide (20 mmol, 2.4 g) was added dropwise. After an additional 30 min, the product was precipitated with water, collected by filtration and recrystallized to give products in 70% yield. Crystals of title compound (0.3 g) were obtained by slow evaporation of an ethanol/water mixture (1:1 v/v, 10 ml).

### Refinement

All H atoms were placed in calculated positions with C—H = 0.93-0.98 Å, and refined with a riding model, U_iso_(H) = 1.2U_eq_(C). The atoms of C20 and C21 are disordered over two sites with 0.5 occupancy for each component.

### Crystal data

**Table:**

C_22_H_20_N_4_	*F*(000) = 720
*M**_r_* = 340.42	*D*_x_ = 1.233 Mg m^−^^3^
Monoclinic, *P*2_1_/*n*	Mo *K*α radiation, λ = 0.71073 Å
Hall symbol: -P 2yn	Cell parameters from 2460 reflections
*a* = 11.008 (2) Å	θ = 3.3–27.5°
*b* = 13.884 (3) Å	µ = 0.08 mm^−^^1^
*c* = 12.540 (3) Å	*T* = 293 K
β = 106.98 (3)°	Block, yellow
*V* = 1833.2 (6) Å^3^	0.30 × 0.25 × 0.22 mm
*Z* = 4	

### Data collection

**Table:**

Rigaku SCXmini diffractometer	2460 reflections with *I* > 2σ(*I*)
Radiation source: fine-focus sealed tube	*R*_int_ = 0.061
graphite	θ_max_ = 27.5°, θ_min_ = 3.3°
Detector resolution: 13.6612 pixels mm^-1^	*h* = −14→14
ω scans	*k* = −17→17
18577 measured reflections	*l* = −16→15
4190 independent reflections	

### Refinement

**Table:**

Refinement on *F*^2^	Primary atom site location: structure-invariant direct methods
Least-squares matrix: full	Secondary atom site location: difference Fourier map
*R*[*F*^2^ > 2σ(*F*^2^)] = 0.058	Hydrogen site location: inferred from neighbouring sites
*wR*(*F*^2^) = 0.149	H-atom parameters constrained
*S* = 1.03	*w* = 1/[σ^2^(*F*_o_^2^) + (0.0589*P*)^2^ + 0.2641*P*] where *P* = (*F*_o_^2^ + 2*F*_c_^2^)/3
4190 reflections	(Δ/σ)_max_ = 0.008
254 parameters	Δρ_max_ = 0.15 e Å^−^^3^
0 restraints	Δρ_min_ = −0.17 e Å^−^^3^

### Special details

**Table:**

Geometry. All esds (except the esd in the dihedral angle between two l.s. planes) are estimated using the full covariance matrix. The cell esds are taken into account individually in the estimation of esds in distances, angles and torsion angles; correlations between esds in cell parameters are only used when they are defined by crystal symmetry. An approximate (isotropic) treatment of cell esds is used for estimating esds involving l.s. planes.
Refinement. Refinement of *F*^2^ against ALL reflections. The weighted *R*-factor wR and goodness of fit *S* are based on *F*^2^, conventional *R*-factors *R* are based on *F*, with *F* set to zero for negative *F*^2^. The threshold expression of *F*^2^ > σ(*F*^2^) is used only for calculating *R*-factors(gt) etc. and is not relevant to the choice of reflections for refinement. *R*-factors based on *F*^2^ are statistically about twice as large as those based on *F*, and *R*- factors based on ALL data will be even larger.

### Fractional atomic coordinates and isotropic or equivalent isotropic displacement parameters (Å^2^)

**Table:**

	*x*	*y*	*z*	*U*_iso_*/*U*_eq_	Occ. (<1)
C1	0.70227 (17)	0.49610 (14)	0.68558 (15)	0.0475 (5)	
C2	0.7143 (2)	0.47856 (16)	0.79793 (16)	0.0606 (6)	
H2A	0.6965	0.4160	0.8224	0.073*	
C3	0.7536 (2)	0.5530 (2)	0.87212 (18)	0.0695 (6)	
H3A	0.7624	0.5425	0.9497	0.083*	
C4	0.7819 (2)	0.64336 (18)	0.83744 (18)	0.0667 (6)	
H4A	0.8079	0.6938	0.8917	0.080*	
C5	0.77246 (18)	0.66223 (16)	0.72750 (18)	0.0607 (6)	
H5A	0.7930	0.7243	0.7036	0.073*	
C6	0.73172 (17)	0.58701 (14)	0.65273 (15)	0.0476 (5)	
C7	0.66687 (17)	0.48863 (13)	0.50843 (15)	0.0463 (5)	
C8	0.63061 (18)	0.45683 (15)	0.39313 (16)	0.0512 (5)	
H8A	0.6325	0.5030	0.3366	0.061*	
C9	0.59503 (17)	0.36809 (14)	0.36025 (15)	0.0497 (5)	
H9A	0.5926	0.3208	0.4154	0.060*	
C10	0.55903 (17)	0.34044 (14)	0.24347 (15)	0.0455 (4)	
C11	0.48500 (17)	0.25237 (14)	0.09206 (15)	0.0485 (5)	
C12	0.4338 (2)	0.18326 (17)	0.01055 (19)	0.0671 (6)	
H12A	0.4073	0.1210	0.0286	0.081*	
C13	0.4604 (2)	0.3003 (2)	−0.12454 (19)	0.0736 (7)	
H13A	0.4532	0.3147	−0.2010	0.088*	
C14	0.5093 (2)	0.36810 (17)	−0.04472 (16)	0.0617 (6)	
H14A	0.5331	0.4308	−0.0640	0.074*	
C15	0.52313 (17)	0.34327 (14)	0.06632 (15)	0.0487 (5)	
C16	0.7238 (2)	0.66083 (15)	0.46662 (17)	0.0606 (6)	
H16A	0.7323	0.6351	0.3981	0.073*	
H16B	0.7996	0.6964	0.5020	0.073*	
C17	0.6124 (2)	0.72847 (16)	0.44099 (16)	0.0595 (6)	
H17A	0.5285	0.7013	0.4194	0.071*	
C18	0.6240 (3)	0.82080 (18)	0.4455 (2)	0.0858 (8)	
H18A	0.7070	0.8494	0.4669	0.103*	
H18B	0.5499	0.8610	0.4285	0.103*	
C19	0.4846 (2)	0.17117 (16)	0.27155 (19)	0.0695 (6)	
H19A	0.4136	0.1329	0.2282	0.083*	
H19B	0.4661	0.1938	0.3383	0.083*	
C20	0.6135 (9)	0.1094 (6)	0.3030 (5)	0.0653 (18)	0.625 (16)
H20A	0.6899	0.1385	0.3413	0.078*	0.625 (16)
C21	0.6122 (10)	0.0200 (6)	0.2758 (5)	0.102 (3)	0.625 (16)
H21A	0.5361	−0.0094	0.2375	0.122*	0.625 (16)
H21B	0.6874	−0.0151	0.2945	0.122*	0.625 (16)
C20'	0.5505 (16)	0.0869 (8)	0.2990 (8)	0.070 (3)	0.375 (16)
H20B	0.5114	0.0332	0.3242	0.084*	0.375 (16)
C21'	0.6665 (16)	0.0799 (17)	0.2913 (11)	0.107 (6)	0.375 (16)
H21C	0.7040	0.1323	0.2672	0.129*	0.375 (16)
H21D	0.7113	0.0225	0.3099	0.129*	0.375 (16)
C22	0.4227 (2)	0.2096 (2)	−0.09844 (19)	0.0763 (7)	
H22A	0.3871	0.1647	−0.1575	0.092*	
N1	0.70924 (15)	0.58089 (11)	0.53845 (13)	0.0495 (4)	
N2	0.66254 (15)	0.43508 (11)	0.59442 (13)	0.0508 (4)	
N3	0.50861 (14)	0.25146 (11)	0.20645 (13)	0.0492 (4)	
N4	0.56959 (15)	0.39678 (12)	0.16199 (13)	0.0519 (4)	

### Atomic displacement parameters (Å^2^)

**Table:**

	*U*^11^	*U*^22^	*U*^33^	*U*^12^	*U*^13^	*U*^23^
C1	0.0431 (10)	0.0553 (12)	0.0437 (11)	0.0080 (9)	0.0123 (8)	−0.0019 (9)
C2	0.0664 (14)	0.0683 (14)	0.0493 (13)	0.0101 (11)	0.0202 (10)	0.0043 (11)
C3	0.0678 (15)	0.0964 (18)	0.0427 (12)	0.0113 (13)	0.0139 (11)	−0.0080 (13)
C4	0.0583 (13)	0.0808 (17)	0.0566 (14)	−0.0005 (12)	0.0097 (10)	−0.0224 (12)
C5	0.0548 (13)	0.0643 (13)	0.0594 (14)	−0.0063 (11)	0.0113 (10)	−0.0127 (11)
C6	0.0397 (10)	0.0556 (12)	0.0455 (11)	0.0020 (9)	0.0092 (8)	−0.0026 (9)
C7	0.0444 (10)	0.0472 (11)	0.0463 (11)	0.0044 (9)	0.0115 (8)	−0.0026 (9)
C8	0.0558 (12)	0.0531 (12)	0.0433 (11)	0.0033 (9)	0.0123 (9)	−0.0003 (9)
C9	0.0508 (11)	0.0540 (12)	0.0436 (11)	0.0039 (9)	0.0124 (9)	0.0024 (9)
C10	0.0440 (10)	0.0477 (11)	0.0443 (11)	0.0011 (9)	0.0121 (8)	−0.0009 (9)
C11	0.0403 (10)	0.0591 (12)	0.0456 (11)	−0.0010 (9)	0.0117 (8)	−0.0089 (9)
C12	0.0574 (13)	0.0779 (16)	0.0658 (15)	−0.0151 (12)	0.0176 (11)	−0.0201 (12)
C13	0.0684 (15)	0.104 (2)	0.0456 (13)	0.0098 (14)	0.0120 (11)	−0.0023 (13)
C14	0.0674 (14)	0.0716 (14)	0.0464 (13)	0.0084 (11)	0.0169 (10)	0.0015 (11)
C15	0.0464 (11)	0.0558 (12)	0.0446 (11)	0.0046 (9)	0.0143 (8)	−0.0021 (9)
C16	0.0659 (13)	0.0631 (13)	0.0562 (13)	−0.0127 (11)	0.0233 (10)	−0.0037 (11)
C17	0.0653 (13)	0.0626 (14)	0.0462 (12)	−0.0072 (11)	0.0095 (10)	0.0053 (10)
C18	0.0754 (17)	0.0703 (17)	0.103 (2)	0.0004 (13)	0.0121 (14)	0.0072 (15)
C19	0.0989 (18)	0.0522 (13)	0.0654 (15)	−0.0098 (13)	0.0364 (13)	−0.0003 (11)
C20	0.077 (5)	0.063 (4)	0.061 (3)	−0.016 (3)	0.028 (3)	0.015 (2)
C21	0.116 (6)	0.075 (5)	0.112 (4)	0.016 (4)	0.031 (4)	−0.003 (3)
C20'	0.080 (8)	0.054 (6)	0.083 (5)	−0.005 (5)	0.037 (5)	0.015 (4)
C21'	0.095 (10)	0.144 (17)	0.097 (7)	0.021 (9)	0.053 (7)	0.004 (9)
C22	0.0629 (15)	0.105 (2)	0.0547 (15)	−0.0058 (14)	0.0068 (11)	−0.0297 (14)
N1	0.0527 (9)	0.0499 (9)	0.0451 (10)	−0.0033 (8)	0.0127 (7)	−0.0031 (8)
N2	0.0559 (10)	0.0492 (9)	0.0473 (10)	0.0033 (8)	0.0149 (8)	−0.0004 (8)
N3	0.0505 (9)	0.0498 (9)	0.0485 (10)	−0.0029 (8)	0.0163 (7)	−0.0027 (7)
N4	0.0586 (10)	0.0524 (10)	0.0451 (10)	−0.0021 (8)	0.0155 (8)	−0.0013 (8)

### Geometric parameters (Å, °)

**Table:**

C1—N2	1.387 (2)	C13—C22	1.395 (3)
C1—C6	1.395 (3)	C13—H13A	0.9600
C1—C2	1.398 (3)	C14—C15	1.399 (3)
C2—C3	1.373 (3)	C14—H14A	0.9599
C2—H2A	0.9600	C15—N4	1.377 (2)
C3—C4	1.392 (3)	C16—N1	1.467 (2)
C3—H3A	0.9601	C16—C17	1.503 (3)
C4—C5	1.377 (3)	C16—H16A	0.9599
C4—H4A	0.9601	C16—H16B	0.9600
C5—C6	1.388 (3)	C17—C18	1.288 (3)
C5—H5A	0.9600	C17—H17A	0.9601
C6—N1	1.384 (2)	C18—H18A	0.9598
C7—N2	1.322 (2)	C18—H18B	0.9600
C7—N1	1.378 (2)	C19—C20'	1.367 (10)
C7—C8	1.452 (3)	C19—N3	1.451 (2)
C8—C9	1.322 (3)	C19—C20	1.605 (8)
C8—H8A	0.9601	C19—H19A	0.9700
C9—C10	1.453 (3)	C19—H19B	0.9700
C9—H9A	0.9598	C20—C21	1.286 (14)
C10—N4	1.319 (2)	C20—H20A	0.9300
C10—N3	1.378 (2)	C21—H21A	0.9300
C11—N3	1.381 (2)	C21—H21B	0.9300
C11—C12	1.395 (3)	C20'—C21'	1.31 (3)
C11—C15	1.397 (3)	C20'—H20B	0.9601
C12—C22	1.385 (3)	C21'—H21C	0.9300
C12—H12A	0.9600	C21'—H21D	0.9300
C13—C14	1.365 (3)	C22—H22A	0.9602
			
N2—C1—C6	110.73 (16)	C17—C16—H16A	109.1
N2—C1—C2	129.69 (19)	N1—C16—H16B	109.9
C6—C1—C2	119.58 (19)	C17—C16—H16B	108.8
C3—C2—C1	118.0 (2)	H16A—C16—H16B	107.9
C3—C2—H2A	121.1	C18—C17—C16	123.2 (2)
C1—C2—H2A	120.8	C18—C17—H17A	118.6
C2—C3—C4	121.4 (2)	C16—C17—H17A	118.2
C2—C3—H3A	119.3	C17—C18—H18A	119.9
C4—C3—H3A	119.2	C17—C18—H18B	120.1
C5—C4—C3	121.7 (2)	H18A—C18—H18B	120.0
C5—C4—H4A	119.4	C20'—C19—N3	129.0 (7)
C3—C4—H4A	118.9	C20'—C19—C20	27.7 (5)
C4—C5—C6	116.6 (2)	N3—C19—C20	104.8 (3)
C4—C5—H5A	121.8	C20'—C19—H19A	87.3
C6—C5—H5A	121.6	N3—C19—H19A	110.8
N1—C6—C5	132.12 (19)	C20—C19—H19A	110.8
N1—C6—C1	105.28 (16)	C20'—C19—H19B	106.9
C5—C6—C1	122.59 (19)	N3—C19—H19B	110.8
N2—C7—N1	112.96 (16)	C20—C19—H19B	110.8
N2—C7—C8	125.19 (18)	H19A—C19—H19B	108.9
N1—C7—C8	121.85 (17)	C21—C20—C19	120.7 (10)
C9—C8—C7	124.36 (19)	C21—C20—H20A	119.7
C9—C8—H8A	117.4	C19—C20—H20A	119.7
C7—C8—H8A	118.2	C20—C21—H21A	120.0
C8—C9—C10	121.97 (18)	C20—C21—H21B	120.0
C8—C9—H9A	118.8	H21A—C21—H21B	120.0
C10—C9—H9A	119.3	C20—C21—H20B	73.3
N4—C10—N3	112.87 (16)	H21A—C21—H20B	65.1
N4—C10—C9	124.38 (17)	H21B—C21—H20B	135.0
N3—C10—C9	122.75 (17)	C21'—C20'—C19	120.1 (19)
N3—C11—C12	131.9 (2)	C21'—C20'—H20B	120.5
N3—C11—C15	105.67 (16)	C19—C20'—H20B	119.5
C12—C11—C15	122.43 (19)	C20'—C21'—H21C	120.0
C22—C12—C11	116.2 (2)	C20'—C21'—H21D	120.0
C22—C12—H12A	121.7	H21C—C21'—H21D	120.0
C11—C12—H12A	122.1	C12—C22—C13	121.5 (2)
C14—C13—C22	122.1 (2)	C12—C22—H22A	119.3
C14—C13—H13A	119.2	C13—C22—H22A	119.2
C22—C13—H13A	118.7	C7—N1—C6	106.49 (15)
C13—C14—C15	117.7 (2)	C7—N1—C16	128.65 (16)
C13—C14—H14A	121.3	C6—N1—C16	124.80 (15)
C15—C14—H14A	121.0	C7—N2—C1	104.53 (16)
N4—C15—C11	110.26 (16)	C10—N3—C11	106.07 (15)
N4—C15—C14	129.74 (19)	C10—N3—C19	128.38 (17)
C11—C15—C14	119.99 (18)	C11—N3—C19	125.55 (17)
N1—C16—C17	112.04 (17)	C10—N4—C15	105.12 (16)
N1—C16—H16A	108.9		
